# Role of Climate Change in Changing Hepatic Health Maps

**DOI:** 10.1007/s40572-022-00352-w

**Published:** 2022-04-28

**Authors:** Amal Saad-Hussein, Haidi Karam-Allah Ramadan, Ashraf Bareedy, Reda Elwakil

**Affiliations:** 1 Department of Environmental and Occupational Medicine, Environment and Climate Change Research Institute, National Research Centre, El-Buhouth Street, Cairo, Dokki Egypt; 2 Department of Tropical Medicine and Gastroenterology, Faculty of Medicine, Assiut University, Assiut, Egypt; 3 Department of Tropical Medicine, Faculty of Medicine, Ain Shams University, Cairo, Egypt

**Keywords:** Climate change, Liver health mapping, Communicable diseases, Non-communicable diseases, Environmental pollutants, Environmental toxins

## Abstract

***Purpose of Review*:**

Climate change (CC) is currently responsible for global weather extremes. These weather extremes could contribute to changes in the pattern of health problems. The purpose of this review is to discuss the role of CC on remapping of hepatic diseases and the mechanisms of re-mapping.

***Recent Findings*:**

CC was found to have a major influence on the distribution and severity of hepatic diseases, such as outbreaks of vector-borne, water or food-borne, parasitic diseases, re-emerging of disappeared diseases, or emerging of new forms of infectious agents. Migration of infected people from endemic areas due to the CC disasters results in rapid dissemination of infectious diseases that leads to outbreaks or endemicity of diseases in new areas. CC could cause increasing chemical emissions, or change in its biodegradability, or restriction in its dispersion, such as PM, PAHs, heavy metals, mycotoxins, and aquatic toxins. Increase in the concentrations of these chemicals may have significant impacts in changing the health map of hepatic toxicity and liver cancer.

***Summary*:**

The current review confirms the role of CC in changing the pattern of several liver health problems and remapping of these problems in several regions of the world. This review could be of high importance to the health decision-makers as an early alarm and prediction of hepatic health problems with the projected CC.

## Introduction

Human activities have proved to cause increased greenhouse gases that warm the atmosphere, land, and ocean. Widespread and rapid changes in the air, soil, ocean, biosphere, and cryosphere have occurred over the past few decades [1]. Climate change (CC) is a global distressing problem which contributes to global burden of diseases and premature deaths. Intergovernmental Panel on Climate Change (IPCC) concluded that, CC, through changing weather patterns, leads to direct and indirect changes in water, air, and food quality, in addition to the changes in ecosystems, agriculture, industry, settlements, and economy [2]. Also, the changes in weather patterns such as temperature changes, precipitation, high frequencies of extreme events, including sea-level rise, proved to influence the appearance and the geographic distribution of many health problems [3].

A lot of recently published evidence indicate that CC has hazardous impacts on the liver health in several aspects. CC may lead to increase in the communicable and non-communicable liver diseases. Global warming has been reported to have an impact on the pathogen selection and resistance, host ecology and immunity, as well as vector ecological niches and capacity, with more potential influence on vector-borne and zoonotic diseases [4]. CC, due to greenhouse gas emissions, affects the rate of reproduction and distribution of the vectors responsible for disease transmission. These changes in the distribution of vectors are suspected to change the distribution of vector-borne diseases in the different locations. CC is associated with deforestation and encroachment into animal habitats, which forced several wild species to migrate and thereby putting these species in close contact with humans and other animals [5]. All these factors could increase the risk and remap the distribution of communicable liver diseases.

Additionally, most of the non-communicable liver diseases could be attributed to the interaction of CC with several factors that affect development, concentration, and dispersion of air pollutants. This interaction could affect the burden of diseases and mortality associated with these pollutants. Global warming increases the risk of development of poor air quality due to the chemical reactions as a result of either biodegradability of some of these chemicals or increases the concentrations of the emissions due to non-biodegradable of others. CC could also lead to restriction of the dispersion of pollutants due to atmospheric stability [6]. Therefore, when these pollutants penetrate into the body either through skin contacts or through inhalation, it will lead to damage of the internal organs or may produce mutations and cancers specifically in the liver and lungs [6].

Furthermore, desertification and climate change expand the global area of dry land [7] and increase the risk of drought [8]. Subsequently, this will increase the risk of frequent exposure to, and resultant adverse health effects of, desert sand dust such as particulate matter which could induce hepatoxocity or hepatic carcinogenesis.

Global warming and the change in the pattern of precipitation will increase the frequency of hepatotoxic cyanobacterial blooms. In addition, CC induces the conditions suitable for the growth of aflatoxigenic producing fungi and mycotoxin production which are carcinogenic to the liver.

## Aim of the Work

This review aimed to discuss the mechanisms of CC. It also informs about the impact of changes in the pattern of temperature, precipitation, and increased extreme events by increasing the frequency of global liver diseases and remapping of some hepatic health problems, including communicable and non-communicable liver health problems.

## Methodology

A literature search was done by authors, which focused on data from peer-reviewed published articles, which contained the keywords of CC and its effects on remapping of the hepatic diseases vulnerable to CC, through PubMed, Google, and Medline searches. Information from non-peer-reviewed sources, such as editorials, letters, websites, and the sources with non-English language, were excluded.

### PubMed Search

A PubMed MeSH search was done using the below keywords, to find the peer-reviewed published articles which discussed the relation between CC and health map of communicable and non-communicable diseases without limitation of time:

Search: (("Climate Change"[Mesh]) OR ("global warming" OR "climatic change" OR "atmospheric change" OR "Meteorological change")) AND (("health map") OR ("emerging disease") OR ("re-emerging disease") OR ("communicable disease") OR ("non-communicable disease") OR ("infectious disease") OR ("endemic disease") OR ("epidemic disease")).

The second step was filtration to include the peer-reviewed published articles that focused on the impacts of CC on liver during without limitation in time as seen below:

Search: ("Climate Change" [MeSH Terms] OR ("global warming" [All Fields] OR "climatic change" [All Fields] OR "atmospheric change" [All Fields] OR "Meteorological change" [All Fields])) AND ("health map" [All Fields] OR "emerging disease" [All Fields] OR "re-emerging disease" [All Fields] OR "communicable disease" [All Fields] OR "non-communicable disease" [All Fields] OR "infectious disease" [All Fields] OR "endemic disease" [All Fields] OR "epidemic disease" [All Fields]) AND 2016/01/01:2021/12/31 [Date - Publication] AND ("liver disease" [All Fields] OR "liver*" [All Fields] OR ("hepatic" [All Fields] OR "hepatics" [All Fields]) OR ("gastroenterology" [MeSH Terms] OR "gastroenterology" [All Fields] OR "hepatology" [All Fields]) OR "hepato*" [All Fields] OR ("hepatitis" [MeSH Terms] OR "hepatitis" [All Fields] OR "hepatitides" [All Fields] OR "hepatitis a" [MeSH Terms] OR "hepatitis a" [All Fields]) OR "hepat*" [All Fields])

The relationship of CC with liver health problems due to exposure to environmental toxins and pollutants did not appear in the previous search; therefore, the below PubMed MeSH search was done, which revealed only one article published in the last 5 years. For that, additional Google and Medline manual searches were done.

For the effect of mycotoxins on the liver, PubMed MeSH search was done, and then filtration of the results to detect the articles discussing the relationship of CC, mycotoxins, and liver:

Search: (("Climate Change"[Mesh]) OR ("global warming" OR "climatic change" OR "atmospheric change" OR "Meteorological change")) AND (fungal mycotoxin) Filters: Free full text,

### Google and Medline Search

Google and Medline searches were done using the keywords of this review focusing on pollutants and toxins vulnerable to various parameters of CC, such as temperature, precipitation, and salinity, which have impacts on the liver. Through manual selection, English peer-reviewed published articles during the last 5 years were included. Articles that did not include effects of any toxins or pollutants sensitive to CC on human liver were excluded.

## Results

The result of the first PubMed MeSH search was 7166 peer-reviewed published articles about climate change and human health during the period between 1976 and 2022. After specifying peer-reviewed published articles that focused on the impacts of CC on liver, 33 peer-reviewed published articles were found during the period between 1991 and 2021.

The second PubMed MeSH search to select the peer-reviewed published articles discussing the relationship of CC on liver health problems caused by exposure to environmental toxins or pollutants revealed that only one article was published in the last 5 years. PubMed MeSH search for the effect of mycotoxins on the liver resulted into 31 articles, and after filtration only five peer-reviewed published articles were detected discussing the role of CC on mycotoxins affecting the liver.

The results of Google and Medline searches and the manual selection of peer-reviewed published articles focusing on the relationships between the various parameters of CC, such as temperature, precipitation salinity, the different toxins and pollutants, and human hepatic health problems, resulted in 10 peer-reviewed published articles.

This review discusses the role of CC on the mapping of some common communicable and non-communicable liver diseases and highlights the importance of considering CC as risk factor for remapping of hepatic diseases and the impacts of CC on the different hepatotoxic or hepato-carcinogenic causative agents mentioned in Table 1. Therefore, the main results from the included articles were presented as communicable and non-communicable liver diseases.

**Table 1:** Studies on the impact of climate changes on different liver diseases

Reference number	Author, year	Country	Risk factors	Target population	Outcome related to liver disease	Comments
[6]	Manisalidis et al., 2020	Global	Climate change (CC)	Particulate matter (PM)	Chemical pollutants including PM concentrations were projected to increase with CC in the atmosphere, that could cause mutations and liver cancers	Narrative Review, CC effects on causative agent
[9]	Tambo et al., 2016	Global	Short-term climate variation	Dengue virus	Change of dengue viral behavior and human-animal-environment interactions due to climatic variations leading to emerge or reemerge the dengue fever	Narrative Review, CC effects on causative agent
[10]	Lindh et al., 2019	Italy	Extreme weather events	1- Chikungunya (CHIKV) isolated from mosquito pools 2- Infected patients	Extreme weather events are favorite for mosquito breeding and causes rapid proliferation of several species of mosquitoes such as *Aedes albopictus,* that spread CHIKV. Outbreaks spread from Indian Ocean to Europe to be registered in Italy causing outbreaks of Italy in 2017, that belongs to a cluster of novel CHIKV viruses originating in India	Cross-section study, CC effects on causative agent, and occurrence of outbreaks
[11]	Calba et al., 2017	France	Climate change (CC)	Chikungunya (CHIKV) infected patients	CC changes of distribution and spread of CHIKV cases with genomic characterization of the virus transmitting by *Aedes albopictus* identified in East-Central South African to France	Cross-section study, CC effects on causative agent
[12]	Paaijmans et al., 2010	Global	Atmospheric daily temperatures, fluctuating temperature, rainfall and humidity	1- Rodent–malaria *Plasmodium chabaudi* 2- Asian malaria vector *Anopheles stephensi*	Atmospheric temperature may either reduce or increase of transmission of malaria, rainfall and the increase in humidity also affect mosquito breeding and survival, leading to the rapid development of infective sporozoites in the mosquitoes	Experimental study, CC effects on causative agent
[13]	Gao et al., 2016	China	Flood events	1- National Notifiable Disease Surveillance System records 2- Yearbook of Meteorological Disasters records	Hepatitis A infections was projected to be increased for the year 2030 to be between 0.382/10^5^ and 0.399/10^5^ due to flood events	Time-series projections study, CC impact on liver disease
[14]	Castelli and Sulis, 2017	Refugee camps	Migration	Refugees	Migration can affect the trends of the eradicated diseases such as cholera and poliomyelitis, and set priorities for action is needed	Review article, CC impact on GIT disease
[15]	Neimanis et al., 2016	Baltic Sea area	Global warming	Seals (marine mammals)	Global warming enhance the occurrence of *Pseudamphistomum truncatum* emerging zoonotic liver trematode in Baltic Sea ecosystem, that is of potential risk for humans	Cross-section study, CC effects on causative agent
[16]	Sonne et al. 2020	Baltic Sea area	Global warming	Baltic grey seals	Global warming can impact the distribution, migration, diet and behavior of marine mammals, and accidental ingestion of contaminated fish with *Pseudamphistomum truncatum* is of potential risk for humans	Narrative Review, CC effects on causative agent
[17]	Caminade et al. 2019	Global	Global warming	*Fasciola hepatica (*pathogens*)*	Global warming became more suitable for the survival and expansion of the free-living cercaria and the intermediate snails, also the increase of grazing season enhanced the exposure of the grazing animals to the parasite, and increasing the possibility of hepatic fascioliasis	Narrative Review, CC effects on causative agent
[18]	Parajuli et al., 2016	Nepal	Climate change (CC)	Rural households	CC may increase toxic environmental chemicals such as biomass combustion associated with heating and cooking needs, that is the major source of household air pollution; such as PM, PAHs, and different gases, these pollutants increase the rate of hepatotoxicity or cancers	Cross-section survey, CC effects on causative agent, and liver diseases
[21]	Zhang et al. 2019	Taiwan	Long-term exposure to ambient fine particulate matter (PM)	Adults	Cross-sectional study used a satellite-based spatio-temporal model to estimate the concentrations of ambient fine PM that causes hapatotoxicity. and shorten the survival of cases of liver cancer	Cross-section study, CC effects on causative agent, and liver diseases
[22]	Benkerroum, 2019	Global	Hot temperature and humid climates	Aflatoxigenic fungi and aflatoxin	Hot and humid climates is the most suitable climate for the growth of aflatoxigenic fungi and aflatoxin production in agricultural products, that may lead to elevation of liver cancers	Narrative Review, CC effects on causative agent
[23]	Wang et al. 2019	Taiwan	Vinyl chloride monomer	School-aged children	Hepatotoxicity was reported in school-aged children living near a vinyl chloride factory	Cross-section study, CC effects on liver diseases
[24]	Anders et al., 2016		vinyl chloride metabolites	C57Bl/6 J mice	Experimental exposure to vinyl chloride metabolites induced significant liver inflammation and injury	Experimental study, CC liver diseases
[25]	Chuang et al., 2020	Taiwan	Vanadium PM_2.5_	female BALB/c mice	Experimental exposure to soluble vanadium, as a soluble metal present in the vicinity of a petrochemical complex, contributed to PM_2.5_ induced oxidative stress in the liver	Experimental study, CC effects on causative agent
[26]	Abdel-Shafy and Mansour, 2016	Global	Precipitation	Polycyclic aromatic hydrocarbons (PAH)	Precipitation is more effective in removing the sorbed PAHs, and contaminate water and food sources. PAH is toxic, mutagenic, and carcinogenic to liver	Narrative Review, CC effects on causative agent
[32]	Nardi et al., 2017	Central Adriatic Sea	Climate change, temperature	Mediterranean mussels, *Mytilus galloprovincialis*	CC influences ecotoxicological effects of environmental contaminants, and the interactions between temperature, pH and Cd had significant effects on responses of the antioxidant system, causing oxidative damages	Experimental study, CC effects on causative agent
[33]	Visser et al., 2012	Netherlands	Temperature, precipitation	Water quality	Future climate scenarios project lower concentrations of Cd and Zn in surface water are projected. The reduced leaching of heavy metals, due to drying of the catchment, showed a positive impact of CC on a limited aspect of surface water quality	The time series predictive study, CC effects on causative agent
[34]	Ciszewski and Grygar, 2016	Global	Climate change and flood	Heavy metals	Flooding due to CC leads to inundation of contaminated land with heavy metals which will be transported in floodwater reaching the freshwater and marine environment and finally to humans	Narrative Review, CC effects on causative agent
[35]	Whitehead et al., 2009	UK	Temperature and heavy rainfall events	Heavy metals	Projected climate change causes increase in atmospheric temperature and high rainfall events that increase resuspension of contaminated suspended sediment and thereby will increase total concentrations of heavy metals with high adsorption capacities to suspended solids in surface waters	Review article, CC effects on causative agent
[36]	Authman et al., 2015	Global	Heavy metals and Climate change	Fish	Global warming and acidification in sea water may increase the methylation rates of hepatotoxic heavy metals. Fish can be used as indicator for water pollution	Narrative Review, CC effects on causative agent
[37]	Manhães et al., 2020	Brazil	Global warming and acidification in sea water	Mercury	Increase the methylation rates of mercury in tuna and tuna-like species	, CC effects on causative agent, and liver diseases
[38]	Kim el al., 2010	China	Soil heavy metal and acid rain	Heavy metals in Chinese cabbage	Phytoavailability of heavy metals were strongly controlled by pH of acid rain and lower pH can elevate the plant uptake of heavy metals, except for Pb. This indicates that acid rain has an adverse effect on surrounding ecosystems	Experimental study, CC effects on causative agent
[39]	Wu et al., 2014	China	Temperature and heavy rainfall frequency	Water quality	In Beijing, the undergoing increased temperature and heavy rainfall frequency affect water quality related to fluoride and arsenic concentrations of most urban lakes, that becoming worse under climate change trend	Cross-section study, CC effects on causative agent
[40]	Dorbac et al. 2016	Serbia	High temperature	Water and fish samples	Sampling of the water and fish (common carp, (*Cyprinus carpio*) was performed. and were found to contain saxitoxin, microcystin, and/or nodularin	Cross-section study, CC effects on causative agent
[41]	Lad et al., 2019		Microcystin-LR (MC-LR)	Mice	Exposure to MC-LR at levels that results in significant exacerbation of hepatic injury	Experimental study, CC effects on causative agent, and liver diseases
[44]	Munkes et al., 2021	Baltic Sea	Global warming	Cyanobacteria (CyanoHAB)	There is a synergism between global warming and eutrophication, that simultaneously intensification of CyanoHAB	Review, CC effects on causative agent
[46]	Lürling et al., 2018	Global	Global warming	Surface water and cyanobacteria	Global warming stimulates growth of hepatotoxic cyanobacteria and increase the cellular toxicity levels directly and indirectly	Experimental study, CC effects on causative agent
[47]	El-Shehawy et al., 2012	Global	Global warming	Cyanobacteria	Global warming effects the physiological and molecular changes in cyanobacteria and resulting effects on hepatotoxin production	Review, CC effects on causative agent
[50]	Liew and Mohd-Redzwan, 2018	Global	Global warming	Mycotoxins	Great evidence that global warming stimulates mycotoxins’ occurrence, and the interactions between gut microbiota and mycotoxins found to play a significant role in the development hepatocellular carcinoma	Review, CC effects on causative agent, and liver diseases
[51]	Leggieri et al., 2021	Global	Climate change and aflatoxins (AFs)	Crops; maize	CC is predicted to increase the risk of AFs contamination in maize, and the improvement of predictive modeling; extension to different crops and geographic areas; and the impact of CC on fungi and mycotoxin co-occurrence, both in crops and their value chains, up to consumers	Systematic review, CC effects on causative agent
[52]	Dövényi-Nagy et al., 2020	Tropical and subtropical geographic regions	Climate change, and mathematical models as risk assessment tools	*A. flavus* and aflatoxins (AFs) in maize	The review focus on the availability of mathematical models as risk assessment tools to predict the possibility of *A. flavus* infection and levels of AF contaminations in maize in a changing climatic environment, and highlights the current agricultural practices used to prevent or, at least, mitigate the deleterious consequences of AFs contaminations	Review, CC effects on causative agent
[53]	Paterson and Lima, 2017	Global	Atmospheric temperature	Thermotolerant and thermophilic fungi	warm areas are more suitable for the growth of fungi producing aflatoxin, but, in hot countries aflatoxin producing fungi will be inhibited	Review, CC effects on causative agent
[54]	Alday et al. 2017	Spain	Precipitation	Amatoxin-containing mushrooms	Movement of mushroom and their fruiting bodies could be used to monitor the impact of early climate changes on forests	Cross setion study, CC effects on causative agent
[55]	Elwakil et al., 2021	Africa	Climate change	Parasitic diseases	Impacts of climate change on the spread of zoonotic vector borne parasitic diseases in Africa	Narrative Review, CC effects on causative agent

### Communicable Diseases

#### Vector-Borne Diseases (VBDs)

Vectors transmitting parasitic or viral diseases are extremely sensitive to CC, as heat waves, extreme weather events, and salinity have direct effects on breeding rates and sites, survival rates and activities of these vectors, and indirect effects on the pathogens such as shortness or elongation of the incubation period inside the vectors. Therefore, CC will change the geographic distribution, intensity of transmission, and seasonality of VBDs [4]. In addition, the uncontrolled movement of livestock and people increases the risk of development of VBD outbreaks such as dengue and Rift Valley hemorrhagic fever [9].

Extreme weather events are favorite for mosquito breeding and cause rapid proliferation of several species of mosquitoes, including *Aedes*,* Culex*, and* Anopheles spp. Aedes aegypti* is a mosquito vector notorious for spreading different viruses causing hepatic complications such as yellow fever, dengue, Rift Valley hemorrhagic fever, chikungunya (CHIKV), and Zika.

CHIKV, as an example, is transmitted through the bite of the infected mosquitoes, mostly *Aedes aegypti* and *Aedes albopictus*. After infection with CHIKV, the virus replicates in many organs including liver and persists in it. Meanwhile, the main reservoir for persistent CHIKV infection is macrophages. CHIKV could also be affected by people and vector movements, as CHIKV outbreaks spread from Indian Ocean to Europe to be registered in Italy [10] and autochthonous cases in France [11].

Malaria transmission is nonlinear as a response to CC. When maximum temperature is close to the upper limit for vector and pathogen, this leads to reduction of transmission, while daily temperature near the minimum boundary increases the transmission. Infection of the liver cells can cause organ congestion, sinusoidal blockage, and cellular inflammation [12]. Moreover, cumulative amounts of rainfall and the increase in humidity affect mosquito breeding and survival, leading to the rapid development of infective sporozoites in the mosquitoes [12].

#### Water and Food-Borne Infectious Diseases

Ambient temperature, precipitation, floods, and humidity may have a significant role in distribution of intermediate hosts and pathogens that cause digestive diseases. In China, severe flood events due to CC were found to be associated with increase in the incidence of hepatitis A viral infections (HAV). Based on the data between 2005 and 2010 and flood event scenarios, the incidence of HAV infections was projected to be increased for the year 2030 to be between 0.382/10^5^ and 0.399/10^5 ^[13]. Moreover, eradicated diseases, such as cholera and poliomyelitis, seemed to be re-emerging in Europe, due to the migration of infected population from their endemic areas [14].

#### Parasitic Diseases

*Pseudamphistomum truncatum* is a newly emerging zoonotic liver trematode affecting Baltic grey seals, with the roach (*Rutilusrutilus*) as a paratenic host. Accidentally, human may ingest fish with metacercariae, which develop to adults in the bile ducts. This accidental ingestion is of potential risk for humans and will be associated with chronic inflammation, fibrosis, and liver failure [15]. Global warming and the development of the toxic algae blooms linked to warm climate will affect the patterns of this infectious disease, which may decrease the risk of these accidental infections to human. In addition, it will impact the distribution, migration, diet and behavior of marine mammals and birds, and the changes in food web dynamics [16].

*Fasciola hepatica* is a real example of the impact of CC on the change in the distribution and the magnitude of parasitic infestations. *Fasciola hepatica* is mainly a sheep liver-fluke but could be transmitted to human if they ingest infested liver. Wet and mild warm climate is essential for the parasitic free-living stages and the intermediate snail host to grow on grassland and needs approximately 3 months for parasite stages to develop from the eggs and for the release of the infective metacercariae [17]. With global warming, the prevalence of infections increased in endemic countries, as climatic conditions became more suitable for the survival and expansion of the free-living cercaria and the intermediate snails; also, the increase of grazing season enhanced the exposure of the grazing animals to the parasite [17].

### Non-Communicable Diseases

The main classes of toxicants of clinical significance discussed in this review are the chemicals that could be affected by CC and have toxic impacts on the liver or/and gastrointestinal tract (GIT), including particulate matter (PM), polycyclic aromatic hydrocarbons (PAHs), and heavy metals, in addition to the mycotoxins including aflatoxins and aquatic toxins such as cyanobacteria. The increase in the concentrations of these chemicals may have significant impacts in changing of the health map of some non-communicable hepatic diseases, such as hepatotoxicity or cancers. Biomass combustion associated with heating and cooking needs is a major source of household air pollution, such as PM, PAHs, and different gases [18].

#### Particulate Matter (PM)

Mostly, PM occurs naturally in the environment as desert dust, forest fire, sea salt, and sulfates from volcanoes; however, PM concentrations were projected to increase with CC in the atmosphere as a result of chemical reactions between the different pollutants due to the global warming, which could cause mutations and cancers of liver and lungs [6]. Artificially, PM is emitted from industrial sources and heavy traffic. The potential shift of the conditions of subtropical desert to higher latitudes is linked to climate change and desertification. PM concentrations in North Africa and the Middle East are among the highest in the world due to dust events [19].

During the 3-day Saharan episode in Texas in 2008, the total dust contribution increased to 64% for PM_2.5_ and 85% for PM_10_ [20]. It was found that long-term exposure to ambient PM_2.5_ air pollution was associated with adverse effects on liver enzymes [21] and shortens the survival of cases of liver cancer. The liver is affected by PM due to the detoxifying role of xenobiotics absorbed from PM [21].

Moreover, aflatoxigenic producing fungi may by carried on PM, which increases hepatic cancers [22]. Hepatotoxicity was reported in school-aged children due to exposure to air pollution in the vicinity of a petrochemical complex [23]. Another animal study found that exposure of C57Bl/6J mice for 24 h to vinyl chloride metabolites induced significant liver inflammation and injury [24]. In a recent study, it was found that soluble vanadium, as a soluble metal present in the vicinity of a petrochemical complex, contributed to PM_2.5_-induced oxidative stress in the liver [25]. However, it remains unclear the effects of released metals from petrochemical industries on hepatotoxicity. This finding suggests potential increase in the risk of hepatotoxicity and carcinogenicity, as PM concentrations were projected to increase with CC [6], due to air pollution episodes that are expected to be associated with stagnation events and heat waves [21].

#### Polycyclic Aromatic Hydrocarbons (PAHs)

PAHs are found in coal and in tar sediments and produced from incomplete combustion of organic matter as in the cases of forest fires, incineration, and engines. Climate change may increase the emission of toxic environmental chemicals from biomass combustion associated with heating and cooking needs [18]. The amounts of PAHs removed from the atmosphere by wet deposition are more easily from the atmosphere than the vapor phase. Thus, precipitation is more effective in removing the sorbed PAHs rather than the vapor phase, while the vapor phase is more efficiently removed from the atmosphere under cold conditions as compared to warm conditions. The removed PAH may contaminate surface water and the soil. Most of the PAH compounds are recognized as toxic, mutagenic, and carcinogenic substances, and long-term exposure may cause liver damage [26]. Therefore, CC may have a significant role in persistence of PAHs in the atmosphere, which may increase the hepatic damaging and carcinogenic effects.

#### The Common Hepatotoxic Heavy Metals

Heavy metals, such as lead, chromium, arsenic, mercury, nickel, and cadmium, may cause hepatotoxicity, in addition to ultrastructural changes in the hepatocytes, that is induced by oxidative stress reactions [27]. Some studies have also reported a relation between chronic arsenic (As) exposure and development of pre-neoplastic liver lesions, abnormal liver function, hepatomegaly, liver sclerosis, fibrosis, cirrhosis, and liver cancer [28]. Several potential mechanisms of hepatocarcinogenesis of As have been proposed, including genotoxicity, generation of free radicals and oxidative stress, disturbance of signal transduction and cellular proliferation, massive alteration in DNA methylation, and direct cytotoxicity; however, the exact mechanism requires further elucidation [29, 30].

Cadmium (Cd), another heavy metal, has been associated with hepatocarcinogenic potential via multiple mechanisms as shown in animal studies [31]. This mandates conducting human case-control studies to compare individuals with cirrhosis and/or HCC with different Cd body burden, perhaps as indicated by urinary Cd. Nardi et al. [32] proved that CC influences ecotoxicological effects of environmental contaminants, due to the interactions between temperature, pH, and Cd had significant effects on induction of metallothioneins and responses of the antioxidant system, causing oxidative damages, which was tissue dependent. But, future climate scenarios in Visser et al. [33] study project lower concentrations of Cd and Zn in surface water. The reduced leaching of heavy metals, due to drying of the catchment, showed a positive impact of CC on a limited aspect of surface water quality.

Repeated occurrence of extreme events as flooding due to climate change leads to inundation of contaminated land with heavy metals which will be transported in floodwater reaching the freshwater and marine environment and finally to human beings [34]. Alternate floods and droughts due to climate changes have been associated with the release of arsenic and contamination of groundwater. Climate change causes increase in air temperature and high rainfall events (high river discharge rates) at some locations which will increase resuspension of contaminated suspended sediment and chemical reactions, thereby will increase total concentrations of heavy metals with high adsorption capacities to suspended solids in surface waters, which leads to deteriorations in the quality of freshwater ecological status [35].

Global warming and acidification in sea water may increase the methylation rates of heavy metals, such as mercury in tuna and tuna-like species [36]. Moreover, soil acidity, resulting from acidic rains, may lead to movement of heavy metals into the watery environment [37, 38]. In Beijing, the undergoing increased temperature and heavy rainfall frequency affect water quality related to fluoride and arsenic concentrations of most urban lakes, which become worse under climate change trend [39].

#### Aquatic Toxins of Cyanobacteria

Freshwater harmful cyanobacterial blooms (CyanoHABs) are recognized to produce a wide range of toxins and bioactive compounds, which are secondary metabolites. The most commonly occurring cyanobacterial toxins are the microcystin and nodularin which are hepatotoxic [40]. These toxins may induce oxidative stress and metabolic disorder [41]. Exposure to microcystins may also alter microRNA (miRNA) expression in the liver and induce liver injury and promote liver tumor [42], which will lead to development of liver cancer, hepatocyte necrosis, cell fragmentation, glycogen depletion, and vacuolization [43].

Climate change plays a significant role in the development of CyanoHABs in fresh water, due to the alteration of temperature and light of the ecosystem that could support the growth of CyanoHAB species. Global warming and the change in the pattern of precipitation will increase the frequency of CyanoHAB, which increases the magnitude and the duration of blooms. Furthermore, high atmospheric temperature leads to increase in the stratification and lowers the viscosity of seawater which is favorable to CyanoHAB growth [40]. Thus, there is a synergism between global warming and eutrophication, which is simultaneous with intensification of CyanoHAB [44]. It has been reported, in a multi-lake analysis, that cyanobacteria appeared more sensitive to the interaction of nutrients and temperature in more eutrophic lakes [45]. Therefore, global warming stimulates growth of hepatotoxic cyanobacteria and increases the cellular toxicity levels directly and indirectly [46].

Therefore, due to CC, there is a projection that the frequency and duration of cyanobacteria bloom exposure will be increasing in the Baltic Sea within the coming years[44], as well as any aquatic area with the similar environmental conditions. This may cause change in the health map and increase the risk of development of hepatic toxicity and carcinogenicity among the coastal residents consuming contaminated water or sea foods with CyanoHAB toxins. Moreover, depletion of dissolved CO_2_ by dense CyanoHAB creates a concentration gradient across the air-water interface [47], which will affect the aquatic ecosystem and the aquatic life and will decrease the protein sources for coastal populations.

#### Fungal Mycotoxins

Mycotoxins are the secondary metabolites produced by certain fungal species including *Aspergillus*, *Penicillium*, and [48]. Mycotoxins of special interest are aflatoxins (AFs), fumonisins (FUMs), and ochratoxinA (OTA). Mycotoxins represent challenges regarding world-wide food safety and embody a substantial economic burden for many countries. Mycotoxins are natural contaminants of agricultural crops, and their prevalence may increase due to global warming [49]. There is a great evidence that global warming stimulates mycotoxins’ occurrence, and the interactions between gut microbiota and mycotoxins were found to play a significant role in the development of hepatocellular carcinoma secondary to mycotoxicosis [50].

Among all mycotoxins, the biggest attention has been given to aflatoxins (AFs), namely AFB1, because it is a well-known human carcinogen, as CC is considered to be an important predictor for the increase of AFs’ risk of crops’ contamination and improvement of predictive modeling, extension to the impact of CC on fungi and mycotoxin co-occurrence, both in crops and their value chains, up to consumers [51]. AFs are secondary metabolites produced by various *Aspergillus* species and infect crops including, e.g., peanut, cotton-seed, maize, nuts, cereals, spices, and dried fruits. AFs are highly liver carcinogenic and can also cause acute toxicity or even be fatal for both livestock and humans if ingested in sufficient amounts; the availability of mathematical models as risk assessment tools for prediction of the possibility of *A. flavus* infection and levels of AFs contaminations in maize within CC highlights the agricultural practices used to prevent or mitigate the deleterious consequences of AF contaminations [52].

In equatorial and sub-equatorial developing countries, traditional agriculture is usually carried out under optimum conditions and suitable climate for fungal growth and aflatoxin production. This situation is further worsened due to CC, which produces conditions increasingly suitable for the growth of aflatoxigenic producing fungi and mycotoxin production [22]. Tropical countries and warm areas are more suitable for the growth of fungi producing aflatoxin, but, in hot countries aflatoxin producing fungi will be inhibited [53]. Changes in climate could result in large fluctuations in the ambient temperature and precipitation that lead to fluctuation in the quantity of aflatoxin producers. These changes in climate are going to have a critical effect on the agricultural sector by altering the climatic conditions for mycotoxigenic fungal growth. That may result in geographical changes in the quantity and the types of mycotoxin producers, specially aflatoxins, that will result in remapping of the health problems resulting from the ingestion of contaminated crops by mycotoxins.

#### Amatoxin-Containing Mushroom

Mushrooms are important forest fungi in many ecosystems, being highly vulnerable to climate change [54]. Amatoxin-containing mushrooms are responsible for most fatal mushroom ingestions with the subsequent delayed-onset hepatocellular necrosis and are produced primarily by 3 species of mushrooms: *Amanita*,* Lepiota*, and *Galerina* [56]. Alday et al. reported that movement of mushroom and their fruiting bodies could be used to monitor the impact of early CC on forests, especially in the Mediterranean region [54].

#### Impacts of CC on liver disease in Africa

Africa is vulnerable to CC due to weak adaptive capacity, high dependence on ecosystem for livestock, and less developed agricultural systems. Expectations of changes in temperature, rainfall patterns, safe water, and man-made ecological changes of irrigation schemes will lead to increase the burden of schistosomiasis, as well as malaria in endemic areas in Africa. CC impacts also the infection control scenario of zoonotic infections including fascioliasis and the expansion of vectors or reservoirs of infection such as visceral leishmaniasis and the other parasitic liver diseases in Africa such as human echinococcosis and amebic liver abscess [55].

## Discussion

The current review discusses the role of CC in changing the distribution of some forms of hepatic health problems, which will lead to change in the global health maps. The objective of this review was to make a focus on the importance of planning health strategies for surveillance and prediction of epidemics that could be associated with the climate disasters, which seem to occur more frequently with CC.

All the above factors led to redrawing of the maps of liver diseases globally. CC in the form of changes in rainfall patterns, global temperature, humidity, and tropical dusty storms may have a major influence on the distribution, features, and severity of communicable and non-communicable liver diseases. Migration of infected people from endemic infection areas due to the CC disasters to other areas is an important way for the rapid dissemination of infectious diseases. Health infrastructures and population density will affect the incidence and prevalence of the controllable infectious diseases. Proper healthcare programs and infrastructures can prevent the development of outbreaks or endemicity of the disease in the new areas.

Proper expectation and designed protective strategies will play a significant role in minimizing the social and economic burdens of the emerging or re-emerging hepatic health problem globally.

Thus, early warning surveillance, monitoring, and mitigation strategies are essential tools to be implemented, besides the sustained mapping and watching of infectious diseases vulnerable to CC to guide the healthcare decision-makers to design health policies according to the risk factors and the indicators for control or elimination programs. Moreover, CC impacts liver by exposure to environmental pollutants sensitive to global warming. CC could also affect the geographical distribution of many infectious diseases, as well as hepatic toxicity and carcinogenicity. Different strategies to improve air quality are needed to decrease these hepatic health problems resulting from exposure to environmental pollutants. These strategies require an estimation of the cost-benefits gained from proposed mitigation programs. Public awareness with the scientific approaches must be considered as an essential target solution to decrease this threat and propose sustainable solutions.

## Conclusion

In conclusion, this review can help policymakers and stockholders in suggesting proper strategies according to the expectations and predicted scenarios of hepatic health impacts due to CC. It can also be used in planning strategies for CC adaptation and mitigation. For the gastroenterologists, this review will develop new vision in the diagnosis and treatment of the hepatological problems. Clinicians should consider CC as a risk factor for several liver diseases and put this into consideration during the management of patients. They should also consider the re-mapping of liver diseases to improve the diagnosis of their patients. For example, considering migration of infected patients from their endemic regions due to the CC would lead to emerging diseases in some areas which will expand the scope of diagnosis of liver diseases. Further researches on CC by hepatologists would be necessary to understand the different mechanisms of inducing liver diseases by CC. This will benefit vulnerable communities to minimize CC risks. Therefore, the review could be of high importance to the health decision-makers as an early alarm for the prediction of hepatic health problems related to CC.
